# Prevalence of and factors associated with unintended pregnancies among sexually active undergraduates in mainland China

**DOI:** 10.1186/s12978-022-01461-3

**Published:** 2022-07-19

**Authors:** Yan Yuan, Fang Ruan, Yusi Liu, Lei Wu, Mingliang Pan, Zijie Ye, Youxiong Zhao, Lu Lin, Li Zhang, Jiajun Liu, Dongsheng Luo, Bangzheng Zhu, Xinyu Liao, Mengsi Hong, Siyi Wang, Jilun Chen, Zihao Li, Gaoming Yang, Hongfang Jiang, Guochen Fu, Junfang Wang

**Affiliations:** 1 Department of Preventive Medicine, Hubei University of Science and Technology, No.88 Xianning Avenue, Xianning City, 437100 Hubei Province China; 2 National Demonstration Center for Experimental General Medicine Education, Hubei University of Science and Technology, No.88 Xianning Avenue, Xianning City, 437100 Hubei Province China

**Keywords:** Undergraduates, Unintended pregnancies, Prevalence, Risk factors

## Abstract

**Background:**

Unintended pregnancies (UIP) among unmarried sexually active college students in mainland China have emerged as a major reproductive health issue with detrimental personal and socioeconomic consequences. This cross-sectional study aimed to determine the prevalence and factors associated with UIP among sexually active undergraduates in mainland China.

**Methods:**

Between September 8, 2019 and January 17, 2020, a total of 48,660 participants were recruited across the Chinese mainland to complete the self-administered, structured, online questionnaire. This analysis was restricted to 6347 sexually experienced, never-married 15–26 year old undergraduates. Pearson’s Chi square tests and multivariate Logistic regression analyses were performed to identify sociodemographic, familial and individual variables associated with UIP.

**Results:**

The overall prevalence of UIP was 17.7%. More specifically, 19.5% of male college students reported they had unintentionally gotten a partner pregnant, while 14.9% of female college students became unintentionally pregnant. Students who experienced UIP were more likely to belong to the older age group (23–26 years), live with only one parent or live without parents at home, report that their family members approve of premarital sex, initiate sexual activity younger than 14 years old and have casual sex partners. Furthermore, females with multiple partners and males who came from low- income households, experienced sexual abuse, perceived difficulties in acquiring condoms and did not know how to use condoms correctly were also at higher risk of experiencing an unintended pregnancy.

**Conclusion:**

In order to prevent UIP, a comprehensive intervention measure should be taken to target older students and those engaging in risky sexual behaviors, work with young male students to improve condom use skills, improve the availability of free condoms, optimize the involvement of parents and other family members in their children’s sex education.

## Plain language summary

In this study, we aimed to determine the prevalence and factors associated with UIP among sexually active undergraduates in mainland China. Between September 8, 2019 and January 17, 2020, a total of 48,660 participants were recruited from the Chinese mainland to complete the self-administered, structured, online questionnaire. This analysis was restricted to 6347 sexually experienced, never-married 15–26 year old undergraduates. Based on a social-ecological theoretical framework, we ran separate multivariate Logistic regression models for men and women to identify sociodemographic, familial and individual variables associated with UIP. Our findings indicate that the overall prevalence of UIP was 17.7%. More specifically, 19.5% of male college students reported they had unintentionally gotten a partner pregnant, while 14.9% of female college students became unintentionally pregnant. Students who experienced UIP were more likely to belong to the older age group, live with only one parent or live without parents at home, report that their family members approve of premarital sex, initiate sexual activity younger than 14 years old and have casual sex partners. Furthermore, females with multiple partners and males who came from low-income households, experienced sexual abuse, perceived difficulties in acquiring condoms and did not know how to use condoms correctly were also at higher risk of having an unintended pregnancy. In order to prevent UIP, a comprehensive intervention measure should be taken to target older students and those engaging in risky sexual behaviors, work with young male students to improve condom use skills, improve the availability of free condoms, optimize the involvement of parents and other family members in their children’s sex education.

## Background

Unintended pregnancies (UIP) are generally defined as the situations in which pregnancies is either mistimed or unwanted at the time of conception [1–4]. Unmarried youth including in-school adolescents in mainland China are more likely to experience UIP, due to lack of knowledge about sexual and reproductive health [5–7], tolerant attitudes toward premarital sex [6–8] and premarital sexual practices [5–9], coupled with non-use or inconsistent use of contraception [7, 9] and the social stigma against premarital sex and pregnancy outside of marriage [4]. For example, Zhou et al. [7] found that 14.4% of respondents were sexually active, of whom more than one-fourth (25.2%) experienced an unintended pregnancy in a large, multi-site sample of college students (n = 74,258). Similarly, a recent survey conducted by Huang, Xiao and Wang [8] indicated that 10.1% of sexually active undergraduates had experienced at least one unintended pregnancy and even a small percentage (1.8%) experienced repeated pregnancies. UIP are not only distressing for the affected woman, but also cause far-reaching medical, social and economic consequences. Therefore, addressing the sexual and reproductive health needs and problems of adolescents is important not only to reduce UIP rates as well as their attendant risks of maternal and perinatal morbidity and mortality, but also to achieve the sustainable development goals (Target 3.7 and Target 5.6) by 2030.

In mainland China, the National Disease Surveillance Points system was built in 2003 and it was well documented that the rate of contraception decreased sharply from 89.1% in 2010 to 80.6% in 2018 due to the introduction of the two-child policy. However, it is noted that these statistics were confined to married women of reproductive age group. Unfortunately, there are currently no published national statistics available about the prevalence and risk factors associated with UIP among unmarried sexually active college students in mainland China. Such information is a prerequisite for efficient development and implementation of the reproductive health program.

### Conceptual framework

According to the social-ecological model, risk factors for UIP can be crudely divided into three levels (individual, familial and social), although the ways in which they were categorized were not consistently reported and varied greatly between different studies [1, 10]. Briefly, individual-level factors, representing the most direct reasons for UIP, comprise risky sexual behaviors (e.g., the history of sexual abuse [11], early sexual debut [11], multiple sexual partners [11]) and the knowledge [2], attitude [12], behaviors [2] and skills related to contraception. Family, as the most proximal and influential context for individual development, is found to be a significant predictor for UIP [10]. Familial risk factors include low education level of parents, economic problems [2, 10], family disruption [10], poor communication between parents and child [10]. Socio- demographic characteristics include gender [13], age [2, 14], area of residence [2, 14], income [14], religion [2, 12], employment [4], and education level [14].

Framed by the social-ecological model (SEM), this study aimed to assess the effects of a wide range of individual, familial and sociodemographic characteristics on UIP amongst never-married sexually active undergraduates aged 15–26 years in mainland China based on a large nationally-distributed sample.

## Methods

### Study design and setting

This web-based cross-sectional survey was carried out between September 8, 2019 and January 17, 2020. The Questionnaire Star (http://www.sojump.com) was chosen as the survey platform due to its simple and user-friendly interface. Prior to conducting the survey, a formal consent was obtained from the Director of Students' Affairs Division and this study also received ethical approval (No. 2021XG001) from the academic ethics and moral supervision committee of Hubei University of Science and Technology (HUSC).

Due to their convenience and better cooperation, students from HUSC were first recruited as participants for the study. Meanwhile, the students were awarded extra course credits, the honour of Outstanding Volunteer and even a certain amount of money to invite their friends and acquaintances to participate in the study. In order to obtain a large, national sample of college students, our research team members also distributed the URLs of this survey to potential participants through emails, instant messages, text messages or other modes of electronic communication. After signing an electronic informed consent form voluntarily, participants completed the questionnaire. Furthermore, participants were also provided with a brief description of the content of the questionnaire, informed that there were not definitely right or wrong answers throughout this questionnaire and assured of the anonymity and confidentiality of their responses. More importantly, all the participants were promised that they could withdraw from the survey at any point if they felt uncomfortable answering any questions.

### Participants

A total of 48,660 completed questionnaires were received. However, the present study was restricted to 6347 undergraduates who must meet the following five inclusion criteria: (a) aged 15–26 years; (b) heterosexuals; (c) never married; (d) sexually active; (e) full-time undergraduates currently registered at one university in mainland China.

### Sample

**Fig. 1:**
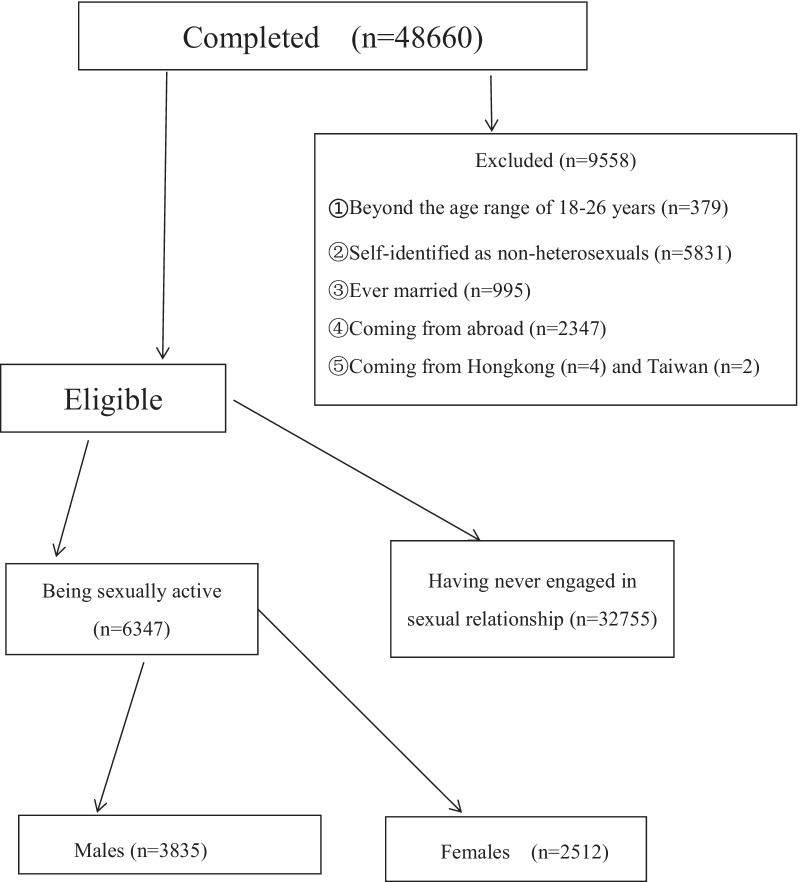
Flow chart showing study sample selection

The details of the study sample selection were shown in Fig. 1. As already mentioned above, the data were extracted from a total of 48,660 completed surveys. 9558 subjects were first excluded because of falling beyond the age range of 18–26 years (n = 379), being identified as a non-heterosexual person (n = 5831), ever being married (n = 995), coming from abroad (n = 2347), Hongkong (n = 4) or Taiwan (n = 2). Then 32,755 respondents who had never engaged in sexual relationship were also excluded. As a result, only 6347 never married, heterosexually active undergraduates aged 15–26 years in mainland China were included into the final analysis.

### Design and content of the questionnaire

The structured questionnaire was developed based on the social-ecological model by the Department of Preventive Medicine, School of Public Health of Hubei University of Science and Technology (HUST), and pilot tested with 50 students conveniently drawn from the selected population.

### The outcome (dependent) variable

Our dependent variable was the UIP experience. Participants were first asked to report whether they had ever engaged in sexual intercourse. And those who gave positive responses were further asked whether they themselves (for females) or their partners (for males) had ever experienced UIP. Given the difficulties in defining UIP, this analysis was further restricted to college students who had never been married [15].

### Explanatory (independent) variables

Based on the existing literature, fourteen variables hypothesized to influence UIP (Table 2) were included in the current analyses. Age was measured as a continuous variable ranging from 15 to 26 (years) and categorized into three groups: (15–17, 18–22, and 23–26). Monthly expenditure in Yuan was used as a proxy to measure family's socioeconomic status (SES) and categorized into low (1 ≤ 1000) and high SES groups (0 ≥ 1000). Living arrangements were classified as follows: living with both parents at home (i.e., intact family), living with only one parent or living without parents at home (i.e., disrupted family) [10]. In addition, the respondents were also asked to indicate their family members’ attitude toward premarital sex on a scale from 1 to 3 (1 = acceptable, 2 = neutral, 3 = unacceptable).

Consistent with previous literature, the experience of sexual abuse was defined as having experienced sexual coercion or violence [11], early sexual debut was defined as having had first sexual intercourse at or before age 14 [11], multiple partners were defined as having had more than two different sexual partners in the past six months [9], and casual sex was defined as having sex with partners without emotional attachment such as commercial sex and one night stand [9].

Four additional variables related to condoms use (i.e., perceived difficulties in acquiring condoms, condom use at first sex, condom use knowledge and skills) were also assessed in this study, due to its continued importance in preventing UIP and HIV/STD infections and also because condoms were the most commonly used contraceptive method accounting for 90.2 percent (5724/6347) of these sexually active undergraduates.

### Statistical analysis

All the data obtained via the website “www.sojump.com” were exported into a Microsoft excel worksheet, double-cleaned, recoded and analyzed independently by the two authors using the Chinese version of SPSS 25.0. The statistical analyses were conducted in the following four steps. Firstly, descriptive statistics were computed for both dependent and independent variables. Secondly, Pearson’s Chi-square tests were used to examine the associations between the dependent variable (i.e., UIP) and each independent variable. After checking for collinearity among independent variables, those variables which were screened using Chi-square tests to have statistically significant associations with UIP were finally entered into multivariable Logistic regression models using backward LR method to control for potential confounders. The impact of multicollinearity was examined by calculating the variance inflation factor (VIF), with a cutoff value of 10 (i.e., VIFs greater than 10 indicate strong multicollinearity). Only variables with a two-tailed P value less than 0.05 were retained in the final model. The adjusted odds ratios (AOR) and 95% confidence intervals (CI) were also reported.

## Results

### Descriptive statistics

Table 1 displayed the provincial distribution of the 6347 sexually experienced undergraduates. As can be seen from Table 1, participants were disproportionately distributed across the Chinese mainland (including 22 provinces, 4 municipalities and 5 autonomous regions), and were mainly recruited from Hubei (32.9%) and Yunan (24.9%).

**Table 1:** Provincial distribution of the study sample (N = 6347)

Region		
Hubei	2087	32.9
Yunan	1578	24.9
Jiangsu	418	6.6
Sichuan	362	5.7
Guizhou	269	4.2
Henan	237	3.7
Hebei	214	3.4
Shanxi	131	2.1
Jilin	113	1.8
Shandong	95	1.5
Shaanxi	87	1.4
Zhejiang	85	1.3
Guangdong	83	1.3
Guangxi	83	1.3
Liaoning	66	1.0
Tianjing	58	0.9
Helongjiang	54	0.9
Gansu	41	0.6
Beijing	38	0.6
Chongqing	38	0.6
Hunan	34	0.5
Shanghai	33	0.5
Inner Mongolia	30	0.5
Anhui	29	0.5
Fujian	21	0.3
Xinjiang	19	0.3
Hainan	16	0.3
Jiangxi	12	0.2
Tibet	7	0.1
Qinghai	6	0.1
Ningxia	3	0.0
Total	6347	100.0

Table 2 provided a summary of the descriptive statistics of the dependent and independent variables. Of 6347 sexually active undergraduates, 1123 reported they had experienced UIP and the overall prevalence of UIP was 17.7% (95% CI: 16.8–18.6%). More specifically, 19.5% (95% CI: 18.5–20.5%) of male college students reported they had unintentionally gotten a partner pregnant, while 14.9% (95% CI: 14.0–15.8%) of female college students became unintentionally pregnant.

**Table 2:** Characteristics of the study sample (N = 6347)

Variable	n	%
Dependent variables		
Unintended Pregnancy		
0 = No	5224	82.3
1 = Yes	1123	17.7
Sociodemographic background		
Gender		
0 = Male	3835	60.4
1 = Female	2512	39.6
Residential areas		
0 = Rural	3257	51.3
1 = Urban	3090	48.7
Age (Years) (Mean = 19.8)		
1 = 15–17	294	4.6
2 = 18–22	5707	89.9
3 = 23–26	346	5.5
Family characteristics		
Monthly expenditure (Yuan)		
0 ≥ 1000	5052	79.6
1 ≤ 1000	1295	20.4
Living arrangements		
0 = Living with both parents	4031	63.5
1 = Living with one parent or Living without parents	2316	36.5
Family attitude toward premarital sex		
0 = Neutral or unacceptable	3955	62.3
1 = Acceptable	2392	37.7
Individual-level		
Experience of sexual abuse		
0 = No or Not sure	5525	87.0
1 = Yes	822	13.0
Age at firs sex		
0 = Older than 14	5851	92.2
1 = Younger than 14	496	7.8
Number of Partner		
0 = Single	4371	68.9
1 = Multiple	1976	31.1
Partner type		
0 = Stable	5971	94.1
1 = Casual	376	5.9
Knowledge about condom use		
0 = Wrong or unsure	1362	21.5
1 = Correct	4985	78.5
Condom use at first sex		
0 = Yes	4152	65.4
1 = No or Not sure	2195	34.6
Difficulties in acquiring condoms		
0 = No difficulty at all	5623	88.6
1 = Some or very difficult	724	11.4
Knowing how to use a condom correctly		
0 = Yes	1033	16.3
1 = No	5314	83.7

As indicated in Table 2, 60.4% were male and 48.7% came from urban areas. The mean age of the participants was 19.8 years and the majority (89.9%) were within the age group of 18–22 years. One-fifth (20.4%) reported that their monthly expenditure was below 1000 Yuan, 36.5% lived with only one parent or lived without parents at home, and 37.7% reported that their family members approved of premarital sex. At the individual level, more than one-tenth (13.0%) had a history of sexual abuse, 7.8% had their first sexual intercourse under 14 years of age, 31.1% reported having multiple sexual partners in the past 6 months, and even a small percentage (5.9%) had sex with casual partners. Although condoms were regarded by nearly four-fifths (78.5%) of respondents as an effective method to prevent UIP and HIV infection, only 65.4% used condoms at first sexual intercourse, 11.4% perceived difficulties in acquiring condoms, and 83.7% did not know how to use condoms correctly (See Table 2).

### Bivariable analysis

Table 3 showed the results from bivariate analysis of the factors associated with UIP among sexually active students by genders. As indicated in Table 3, eight independent variables, including age, monthly expenditure, living arrangements, family’s attitude toward premarital sex, experience of sexual abuse, age at firs sex, partner type and perceived difficulties in acquiring condoms were found to be significantly associated with UIP among both males and females. Furthermore, three variables (i.e., residential areas, condom use at first sex and condom use skills) were only significantly associated with UIP among males, while number of sexual partners was only significantly associated with UIP among females. In addition, condom use knowledge showed no significant association with UIP among either males or females.

**Table 3:** Bivariate analysis of factors with unintended pregnancy among sexually active students by genders

Variable	Males (n = 3835)			χ^2^	p	Females (n = 2512)			χ^2^	p
	Total	Pregnancy	%			Total	Pregnancy	%		
Residential areas										
0 = Rural	1886	342	18.1	4.61	0.032	1371	198	14.4	0.48	0.491
1 = Urban	1949	407	20.9			1141	176	15.4		
Age (Years)										
1 = 15–17	210	49	23.3	24.54	< 0.001	84	13	15.5	36.84	< 0.001
2 = 18–22	3387	626	18.5			2320	323	13.9		
3 = 23–26	238	74	31.1			108	38	35.2		
Monthly expenditure (Yuan)										
0 ≥ 1000	3056	452	14.8	215.08	< 0.001	1996	273	13.7	11.25	0.001
1 ≤ 1000	779	297	38.1			516	101	19.6		
Living arrangement										
0 = Living with both parents	2297	347	15.1	71.33	< 0.001	1734	222	12.8	19.22	< 0.001
1 = Others*	1538	402	26.1			778	152	19.5		
Family’s attitude toward premarital sex										
0 = Neutral or unacceptable	2046	347	12.3	147.21	< 0.001	1909	211	11.1	92.33	< 0.001
1 = Acceptable	1789	402	27.8			603	163	27.0		
Experience of sexual abuse										
0 = No or Not sure	3493	524	15.0	511.25	< 0.001	2032	276	13.6	14.31	< 0.001
1 = Yes	342	225	65.8			480	98	20.4		
Age at firs sex										
0 = Older than 14	3478	454	13.1	997.35	< 0.001	2373	275	11.6	368.51	< 0.001
1 = Younger than 14	357	295	82.6			139	99	71.2		
Number of Partners										
0 = Single	2533	497	19.6	0.04	0.844	1838	228	12.4	33.35	< 0.001
1 = Multiple	1302	252	19.4			674	146	21.7		
Partner type										
0 = Stable	3545	646	18.2	51.02	< 0.001	2426	332	13.7	80.99	< 0.001
1 = Casual	290	103	35.5			86	42	48.8		
Condom use knowledge										
0 = Wrong or unsure	725	160	22.1	3.67	0.056	637	96	15.1	0.02	0.881
1 = Correct	3110	589	18.9			1875	278	14.8		
Condom use at first sex										
0 = Yes	2487	510	20.5	4.29	0.038	1665	234	14.1	2.71	0.099
1 = No or Not sure	1348	239	17.7			847	140	16.5		
Difficulties in acquiring condoms										
0 = No difficulty at all	3448	631	18.3	32.90	< 0.001	2175	305	14.0	9.59	0.002
1 = Some or very difficult	387	118	30.5			337	69	20.5		
Condom use skills										
0 = Knowing how to use condoms	822	111	13.5	24.18	< 0.001	211	37	17.5	1.27	0.259
1 = Not knowing how to use condoms	3013	638	21.2			2301	337	14.6		

### Multicollinearity

As indicated in Table 4, all VIF values ranged between 1.01 and 1.26, which is well below the widely used threshold of 10, indicating the absence of multicollinearity.

**Table 4:** Multicollinearity diagnosis for the linear regression

Variable	Males (n = 3835)		Females (n = 2512)	
	Tolerance	VIF	Tolerance	VIF
Residential areas	1.00	1.01	0.97	1.03
Age (Years)	0.95	1.05	0.93	1.07
Monthly expenditure (Yuan)	0.89	1.12	0.96	1.04
Living arrangements	0.96	1.05	0.97	1.03
Family attitude toward premarital sex	0.95	1.05	0.95	1.06
Experience of sexual abuse	0.82	1.22	0.97	1.03
Age at firs sex	0.79	1.26	0.88	1.13
Number of Partner	0.94	1.06	0.96	1.04
Partner type	0.95	1.06	0.90	1.11
Knowledge about condom use	0.98	1.03	0.98	1.03
Condom use at first sex	0.96	1.05	0.96	1.05
Difficulties in acquiring condoms	0.97	1.04	0.96	1.04
Knowing how to use a condom correctly	0.97	1.03	0.98	1.02

### Multivariate logistic regression analyses

Separate Logistic regression analyses were finally performed to identify statistically significant variables affecting UIP among males and females. As indicated in Table 5, five risk factors related to UIP were identified among both males and females. More specifically, those who experienced UIP were more likely to belong to the older age group (23–26 years) (AOR = 1.76, 95% CI:1.24–2.51; AOR = 2.16, 95% CI:1.33–3.51, respectively), live with only one parent or live without parents at home (AOR = 1.33, 95% CI:1.09–1.62; AOR = 1.35, 95% CI:1.04–1.74, respectively), report that their family members approved of premarital sex (AOR = 2.07, 95% CI:1.70–2.52; AOR = 2.25, 95% CI: 1.75–2.91, respectively), initiate sexual activity younger than 14 years old (AOR = 16.79, 95% CI:12.28–22.96; AOR = 12.64, 95% CI: 8.38–19.07, respectively) and have casual sex partners (AOR = 1.73, 95% CI:1.25–2.38; AOR = 2.49, 95% CI:1.46–4.24, respectively). Furthermore, females with multiple partners (AOR = 1.92; 95% CI:1.49–2.49) and males who came from low-income households (AOR = 1.94; 95% CI:1.55–2.43), experienced sexual abuse (AOR = 4.62; 95% CI:3.41–6.26), perceived difficulties in acquiring condoms (AOR = 1.60; 95% CI:1.19–2.14) and did not know how to use condoms correctly (AOR = 1.34; 95% CI: 1.04–1.72) were more likely to experience UIP.

**Table 5:** Multivariable analysis of factors associated with unintended pregnancy among sexually active students by genders

Variable	Males (n = 3835)		Females (n = 2512)	
	Adjusted OR	95% CI	Adjusted OR	95% CI
Age (0 = 15–17, 1 = 23–26)	1.76**	1.24–2.51	2.16**	1.33–3.51
Monthly expenditure (Ref: ≥ 1000Yuan)	1.94***	1.55–2.43		
Living arrangement (Ref: Living with both parents)	1.33**	1.09–1.62	1.35*	1.04–1.74
Family attitude toward premarital sex (Ref: Neutral or unacceptable)	2.07***	1.70–2.52	2.25***	1.75–2.91
Experience of sexual abuse (0 = No or Not sure, 1 = Yes)	4.62***	3.41–6.26		
Age at firs sex (Ref: Older than 14)	16.79***	12.28–22.96	12.64***	8.38–19.07
Number of Partner (Ref: Single)			1.92***	1.49–2.49
Partner type (Ref: Stable)	1.73***	1.25–2.38	2.49***	1.46–4.24
Difficulties in acquiring condoms (Ref: No difficulty at all)	1.60**	1.19–2.14		
Knowing how to use condoms (0 = Yes, 1 = No)	1.34*	1.04–1.72		

*P < 0.05, **P < 0.01, and ***P < 0.001

## Discussion

### Main findings of this study

In this cross-selectional study, the UIP prevalence among unmarried sexually active college students aged 15–26 years old was 17.7%, which is higher than the rate of 10.1 percent reported by Huang, Xiao and Wang (2020) [8], but lower than the overall level calculated by Zhou et al. (2009) [7]. The discrepancy may be partially attributable to differences in measurement [16] and differences in demographic and socioeconomic characteristics of the sampled participants [4, 16], but may also indicate a decreasing trend in UIP prevalence over time [17]. For example, the global annual UIP rate was 79 per 1000 women aged 15–49 years in 1990–1994, and the number decreased by 15 points to 64 in 2015–2019, according to the most recent figures estimated by Bearak et al. [17].

Based on the social-ecological model, multivariate Logistic regression analysis revealed that, for both males and females, UIP was significantly associated with older age (23–26 years), living with only one parent or living without parents, reporting that their family members approved premarital sex, initiating sexual activity younger than 14 years old and having a casual sex partner. Furthermore, females with multiple partners and males who came from low-income households, experienced sexual abuse, perceived difficulties in acquiring condoms and did not know how to use condoms correctly were also at higher risk of having UIP.

### Comparisons with previous studies

Undergraduates with UIP were more likely to come from broken families and report that their family members approve of premarital sex. This finding can be partly explained by two facts. One possible explanation is that those from broken families had lower perception of family strengths and therefore were less likely to feel satisfied with the quality of their communication with parents, thus contributing to the formation of anxiety, inferiority, insecurity and loneliness which were usually compensated for by various defense mechanism such as entering into a heterosexual relationships or conceiving a baby [10]. The other possible explanation is that families with more tolerant attitudes toward premarital sex exercised little supervision over their children’s dating relationships and might unwittingly push their children towards irresponsible sexual behavior [10]. Furthermore, male students from low-income households were also found to be more likely to report UIP, consistent with previous studies [2, 10, 13, 14, 18]. This finding is not surprising because those from low-income households [2, 14] often gain less knowledge about sexuality and contraception, know less about the availability of free contraceptives and have limited ability to afford modern contraceptives such as condoms, injectable hormones and oral pills, thus contributing to the occurrence of UIP.

The history of sexual abuse [11], having multiple [13, 15] and casual sexual partners [13] have been well recognized as key risk factors for UIP. Consistent with the finding of a survey conducted by Calvert et al. [15], older age and early sexual debut were also significantly associated with increased odds of UIP. This could be due to the fact that early sexual debut and older age might increase both the possibility of engaging in risky sex (i.e., discontinuation, incorrect use and inconsistent use of contraceptives) and the odds of becoming pregnant, thus leading to the occurrence of UIP.

This finding is consistent with previous research that has found no association between knowledge of sexual health and UIP [15]. This phenomenon can be explained by at least two reasons. First, a one-item question to measure respondents' knowledge about pregnancy prevention methods focused only on condom-use and not on any other contraceptive method [15]. Second, condom-use knowledge might exert indirect effects [15] through skills or self-efficacy captured in this study such as condom-use skills and perceived difficulties in acquiring condoms.

### Limitations

Several limitations of this study should be taken into consideration. First, because this study was cross-sectional in nature, the cause-effect relationship between the probability of UIP and a range of familial, demographic and individual factors cannot be established. Second, nonrandom sampling procedure in the present study might produce biased parameter estimates due to the lack of representativeness of the sample and thus limit the generalizability or external validity of the results. Third, data on sexuality and UIP were obtained by self-report and might be subject to recall and social desirability bias. Fourth, due to lack of couple-level data [18, 19], the agreement between the females' self- and partner-reports of experiencing UIP was not assessed in this study. Finally, other potential factors which have not been studied extensively include unhealthy behaviours such as smoking, drinking and drug abuse [16], partners and peers [1], community characteristics (e.g., community media exposure, community fertility norm and community education) [3], as well as policy or relevant legislation [1].

### Implications of the study

Our findings have several important implications. First, target older students and those engaging in risky sexual behaviors. Older age, early sexual debut and sexual abuse were found to be significantly associated with an increased probability of engaging in risky sexual behaviors and experiencing UIP. Furthermore, correct and consistent condom use can prevent both UIP and HIV/STD infections. Therefore, the 100% Condom Use Program should immediately be promoted to target students with these characteristics.

Second, work with male students to improve condom-use skills and improve the availability of free condoms. Males who came from low-income households, perceived difficulties in acquiring condoms and did not know how to use condoms correctly were found to have a higher proportion of UIP. In order to promote condom use, the first and foremost intervention is to work with male students to improve their condom-use skills [20]. Also, intervention should focus on identifying barriers to condom acquisition and delivering free condoms to male students (especially those economically disadvantaged).

Third, optimize the involvement of parents and other family members in their children’s sex education. Our study showed that approximately 40 percent of the students came from broken families, and adverse family events such as parental absence, parental separation or divorce might result in inadequate care and support and potentially contributed to the occurrence of UIP. Furthermore, individuals who report that their family members approve of premarital sex were more likely to experience UIP. Therefore, students’ family members, especially their parents must be involved in educational programs to foster their values related to responsible sexual behavior and wise decision making [21–24]. An optimal family centered approach is expected to create an environment where parents communicate with their adolescent children about sexual issues more frequently and with greater ease [21–24].

## Conclusions

To the best of our knowledge, ours is the first to estimate the prevalence of UIP and associated factors in a large undergraduate sample across the Chinese mainland as well as to assess whether these determinants differ between males and females based on the social-ecological model. Our findings suggested the overall level of UIP has remained high among sexually experienced undergraduates in mainland China. Furthermore, undergraduates who experienced UIP were more likely to belong to the older age group (23–26 years), live with only one parent or live without parents at home, report that their family members approve of premarital sex, initiate sexual activity younger than 14 years old and have casual sexual partners. In addition, females with multiple partners and males who came from low-income households, experienced sexual abuse, perceived difficulties in acquiring condoms and did not know how to use condoms correctly were also at higher risk of experiencing UIP. In order to prevent UIP, a comprehensive intervention measure should be taken to target older students and those engaging in risky sexual behaviors, work with young male students to improve their condom use skills, improve the availability of free condoms, optimize the involvement of parents and other family members in their children’s sex education.

## Data Availability

All data generated or analyzed during this study are included in this published article. Besides, all other data supporting the findings of this study are available from the corresponding author on a reasonable request.

## Abbreviations

UIP: Unintended pregnancies
HUSC: Hubei University of Science and Technology
CCU: Consistent condom use
AOR: Adjusted odds ratio
CI: Confidential interval
HIV: Human immune deficiency virus
STD: Sexually transmitted diseases
